# A nurse-led bundled care intervention for preventing deep vein thrombosis in immobilized neurosurgical patients: a quasi-experimental study

**DOI:** 10.3389/fneur.2026.1842917

**Published:** 2026-06-01

**Authors:** Li Chen, Longfen Zhou, Ju Huang, Mingjie Chen

**Affiliations:** Medical Affairs Department, Xichang People’s Hospital, Liangshan, Sichuan, China

**Keywords:** bundled care, deep vein thrombosis, immobilization, neurosurgery, nurse-led, venous thromboembolism

## Abstract

**Background:**

Immobilized neurosurgical patients are at high risk for deep vein thrombosis (DVT). While routine prophylaxis exists, a systematic, nurse-led bundle of care may offer a more comprehensive and effective approach to prevention. To evaluate the effectiveness of a nurse-led, multifaceted bundled care intervention on the incidence of DVT, patient compliance, and clinical outcomes in immobilized neurosurgical patients.

**Methods:**

This quasi-experimental study with a historical control group was conducted in the Department of Neurosurgery, Xichang People’s Hospital. Adult neurosurgical patients requiring immobilization for at least 72 h were enrolled. The control group (*n* = 1,163, recruited January 2021–September 2022) received standard neurosurgical care. The intervention group (*n* = 1,625, recruited January 2023–December 2024) received a nurse-led bundled care protocol consisting of: (1) dynamic Caprini risk assessment; (2) standardized mechanical prophylaxis with graduated compression stockings (GCS) and intermittent pneumatic compression (IPC); (3) structured health education; and (4) individualized early mobilization. The primary outcome was symptomatic ultrasound-confirmed DVT incidence. Secondary outcomes included compliance, DVT-related knowledge, limb circumference difference, pain scores (Visual Analogue Scale, VAS), and patient satisfaction. Multivariable logistic regression was used to adjust for potential confounders.

**Results:**

Baseline characteristics were comparable between groups. The incidence of DVT was significantly lower in the intervention group (1.66% vs. 2.75%, *p* = 0.041). Intervention group patients demonstrated significantly higher compliance with GCS/IPC use (76.5% vs. 58.1%, *p* < 0.001) and early mobilization (72.1% vs. 51.5%, *p* < 0.001), as well as significantly greater DVT-related knowledge (85.2 ± 8.5 vs. 62.1 ± 10.2, *p* < 0.001). The intervention group showed significantly greater reduction in limb circumference difference (1.2 ± 0.8 cm vs. 2.1 ± 1.1 cm, *p* < 0.001) and lower pain scores (2.3 ± 1.2 vs. 4.1 ± 1.6, *p* < 0.001). Patient satisfaction was significantly higher in the intervention group (94.1 ± 3.3 vs. 85.6 ± 4.8, *p* < 0.001). Pulmonary embolism (PE) incidence was low in both groups (0.12% vs. 0.17%, *p* = 0.715). After adjustment for age, sex, diagnosis, type of surgery, Caprini score, pharmacological prophylaxis, and calendar year, the intervention remained independently associated with lower DVT risk (adjusted OR = 0.54, 95% CI 0.32–0.92, *p* = 0.023).

**Conclusion:**

This nurse-led bundled care intervention was associated with a significantly lower DVT incidence and improved clinical outcomes in immobilized neurosurgical patients. The protocol’s structured, evidence-based approach provides a practical model for VTE prevention in neurosurgical settings.

## Introduction

Deep vein thrombosis (DVT) is a prevalent and potentially life-threatening complication in neurosurgical patients, particularly those who are immobilized due to their condition or post-operative recovery (1, 2). The triad of venous stasis, endothelial injury, and hypercoagulability is frequently observed in this population, stemming from factors such as prolonged surgery, tissue damage, and post-operative immobility. Previous studies have reported DVT incidence rates ranging from 15 to 40% in neurosurgical patients without prophylaxis, and 5 to 15% with standard prophylaxis (3). A recent large-scale review by Banerjee et al. examining patients in neurocritical care unit (including 669,725 individuals with traumatic brain injury, intracerebral hemorrhage, and spinal cord injury) confirmed that this population remains at substantially elevated venous thromboembolism (VTE) risk, with early pharmacological prophylaxis demonstrating significant benefits when initiated within 24–72 h.

Traditional prophylactic approaches, often consisting of single interventions like anticoagulants or basic mechanical measures, have limitations (4). Anticoagulants carry a risk of bleeding, a major concern in neurosurgery, a challenge highlighted by Banerjee et al. (5) who noted the ongoing debate regarding optimal timing of pharmacologic thromboprophylaxis to balance VTE prevention against bleeding complications. Meanwhile, patient compliance with mechanical devices and simple activity recommendations can be poor, often resulting in suboptimal prevention (6). At our institution, despite high VTE risk assessment rates (99.8%), the hospital-associated VTE rate remained around 0.3% from 2021 to 2024, suggesting a gap between risk identification and effective prevention implementation. This gap is particularly relevant for immobilized neurosurgical patients, who represent one of the highest-risk subgroups within the hospital population.

A bundled care approach, which integrates several evidence-based interventions delivered consistently and collectively, has emerged as a more powerful strategy than individual components alone (7). By targeting multiple pathophysiological mechanisms, a bundle aims to create a synergistic effect, leading to significantly improved patient outcomes. Recent evidence supports the effectiveness of bundled interventions in reducing VTE in neurosurgical populations, with consistent effects observed across different settings (8, 9).

Nurses, as the primary caregivers at the bedside, are ideally positioned to lead such an initiative. They can drive continuous risk assessment, ensure the correct application of mechanical devices, deliver patient education, and champion early mobilization (10). Recent evidence from the GRACE trial protocol (11), a large UK multicenter randomized controlled trial involving 8,608 surgical patients, is actively investigating the adjuvant benefit of graduated compression stockings (GCS) in addition to extended pharmacological thromboprophylaxis, underscoring the continued international interest in optimizing mechanical prevention strategies. Furthermore, a quality improvement initiative at Ochsner Health System reported that perioperative VTE incidence in neurosurgical patients receiving chemoprophylaxis consistently ranges between 1.7 and 3.5%, highlighting both the persistent risk and the need for standardized protocols in this population (12). Hazeltine et al. (13) demonstrated the effectiveness of Caprini risk assessment combined with nursing interventions in reducing DVT events in neurosurgical patients, supporting the feasibility of nurse-led approaches in this setting.

Given the high baseline DVT risk in immobilized neurosurgical patients, the documented gap between risk assessment and effective prevention at our institution, and the growing evidence supporting nurse-led bundled interventions internationally, we designed and implemented a comprehensive nurse-led bundled care protocol. This protocol integrates four core components: dynamic Caprini risk assessment, standardized mechanical prophylaxis (GCS and intermittent pneumatic compression [IPC]), structured multi-stage health education, and individualized progressive early mobilization. Therefore, this study aimed to evaluate the association between a comprehensive, nurse-led bundled care intervention and DVT incidence and clinical outcomes in immobilized neurosurgical patients at a tertiary hospital in China.

## Methods

### Study design

A quasi-experimental study design with a historical control group was employed. This design was chosen to implement a systematic change in clinical practice without the risk of contamination between groups inherent in a randomized controlled trial within a single ward. The study was conducted in the Department of Neurosurgery, Xichang People’s Hospital, a large tertiary care center in Southwest China. The study was approved by the Ethics Committee of Xichang People’s Hospital. All participants or their legal guardians provided written informed consent. Patients in the control group were assured that their care would not be compromised by their participation. Patient data were anonymized to ensure confidentiality.

### Participants

#### Inclusion criteria

Patients were included if they: (1) were aged ≥ 18 years; (2) had a confirmed neurosurgical diagnosis (e.g., traumatic brain injury, brain tumor, spinal cord injury, cerebrovascular disease); (3) were prescribed bed rest or immobilization for a minimum of 72 h; and (4) provided written informed consent from themselves or their legal guardians.

#### Exclusion criteria

Patients were excluded if they: (1) had a pre-existing DVT or pulmonary embolism (PE) upon admission, confirmed by Doppler ultrasound; (2) had active bleeding or high bleeding risk contraindicating mechanical prophylaxis; (3) had severe peripheral vascular disease or lower limb skin conditions preventing the use of GCS or IPC; (4) were pregnant or lactating; or (5) had a life expectancy of less than 3 months.

### Recruitment and group allocation

A consecutive sample of eligible patients was recruited. The control group was recruited between January 2021 and September 2022 and received standard care. A 3-month dedicated washout and training period (October–December 2022) followed, during which no new patients were enrolled into the control group. The intervention group was recruited between January 2023 and December 2024 and received the nurse-led bundled care protocol.

### Interventions

#### Control group (standard care)

Patients received routine neurosurgical nursing care. Pharmacological thromboprophylaxis was prescribed at the discretion of the attending physician; when used, it typically consisted of enoxaparin 40 mg subcutaneously once daily, initiated 24–72 h post-surgery if hemostasis was assured. GCS and IPC were available but were applied on a non-protocolized basis (i.e., without mandatory daily checks or standardized usage targets). General health education regarding early mobilization and leg exercises was provided verbally by bedside nurses, but no structured educational curriculum or teach-back method was employed. The hospital-wide VTE prevention rate for high-risk patients during the control period was 60.5%.

#### Intervention group (nurse-led bundled care)

A comprehensive, four-component bundle, designed and led by a dedicated team of trained neurosurgical nurses, was implemented. The bundle was initiated upon admission and continued throughout the hospital stay.

Component 1: Dynamic Risk Assessment using Caprini RAM:

The Caprini Risk Assessment Model was used by the responsible nurse to assess patients within 24 h of admission, post-operatively, and upon any significant change in condition. Risk levels (low, moderate, high, highest) were determined and recorded in the electronic health record (EHR), guiding the intensity of subsequent interventions.

Component 2: Standardized Mechanical Prophylaxis:

GCS: For moderate-to-high risk patients, nurses measured patients’ legs to select the correct size and type of GCS. Patients/families were taught to wear them correctly (applied in the morning, removed at night), and nurses checked skin integrity and proper fit daily.

IPC: For high and highest-risk patients, IPC devices were applied in addition to GCS. Nurses ensured correct placement, set appropriate pressure cycles (35–50 mmHg with a 1:1 to 1:2 compression cycle), and ensured a minimum daily usage time of 18 h. Device function and patient skin were monitored hourly.

Component 3: Structured and Multi-Stage Health Education:

A structured education program was delivered at key time points (admission, pre/post-operation, pre-discharge). Content included: DVT risks, signs and symptoms (e.g., unilateral leg swelling, pain), the purpose of each bundle component, and the importance of patient compliance. Methods included face-to-face teaching, informational booklets, videos, and demonstrations. Teach-back methods were used to confirm patient understanding.

Component 4: Individualized and Progressive Early Mobilization:

Collaborating with the medical team and physiotherapists, nurses initiated a graded activity plan as soon as medically safe (often 6–24 h post-surgery). The plan progressed from: (1) passive/active bed exercises (ankle pumps, quadriceps sets) to (2) sitting on the edge of the bed, (3) standing with assistance, and (4) ambulation. Activity type, frequency, and duration were documented daily.

### Outcome measures

#### Primary outcome

Incidence of symptomatic DVT: Confirmed by compression duplex ultrasound of the lower extremities, performed based on clinical suspicion (e.g., swelling, pain, tenderness, Homans sign).

#### Secondary outcomes

Compliance with Prophylaxis: Rates of correct GCS/IPC usage (recorded via daily nurse checks and device usage meters) and completion of early mobilization activities (recorded in patient charts).

Patient knowledge: Assessed using a validated self-designed questionnaire (Cronbach’s *α* = 0.86) before the intervention (for control group, at a comparable time point) and at discharge. The questionnaire covered DVT risk factors, symptoms, and prevention methods, with a maximum score of 100.

##### Clinical symptoms

*Limb Circumference Difference:* Measured daily at 10 cm above and below the patella using a standardized measuring tape.

*Pain:* Assessed using a Visual Analogue Scale (VAS, 0–10) for pain.

Patient Satisfaction: Measured at discharge using a validated hospital satisfaction survey (0–100 scale).

Safety Outcomes: Incidence of PE (suspected and confirmed), bleeding events, and skin complications related to mechanical devices.

### Data collection

Data were collected by trained research nurses who were not directly involved in patient care. Baseline demographic and clinical data were collected from medical records. Outcome data were collected at pre-defined time points using standardized forms. Detailed information on pharmacological thromboprophylaxis (type, dose, initiation day, and duration) was extracted from the medication administration records for all participants in both periods. All ultrasound examinations were performed by experienced radiologists blinded to group allocation.

### Statistical analysis

Data were analyzed using SPSS version 26.0. Baseline comparisons were performed using independent t-tests or Mann–Whitney *U* tests for continuous variables, and chi-square or Fisher’s exact tests for categorical variables. As a primary analysis, unadjusted comparisons of DVT incidence and secondary outcomes were conducted using chi-square tests. To account for the non-randomized design, we performed multivariable logistic regression with symptomatic DVT as the dependent variable. Covariates included group (intervention vs. control), age (continuous), sex, diagnostic category, type of surgery (cranial, spinal, non-surgical), Caprini score (continuous), use of pharmacological thromboprophylaxis (yes/no), and calendar year (2021, 2022, 2023, 2024 as a continuous ordinal variable to adjust for secular trends). Adjusted odds ratios (aOR) with 95% confidence intervals (CIs) were calculated. A sensitivity analysis was conducted by excluding patients who received any form of pharmacological prophylaxis to assess the independent contribution of the mechanical and educational bundle components. Subgroup analyses of the primary outcome were performed using stratified analysis; the Mantel–Haenszel risk ratio (RR) with 95% CI was calculated. No time-to-event or survival analysis was used. A two-tailed *p*-value of <0.05 was considered statistically significant.

### Ethical considerations

The study was approved by the Ethics Committee of Xichang People’s Hospital. All participants or their legal guardians provided written informed consent. Patients in the control group were assured that their care would not be compromised by their participation. Patient data were anonymized to ensure confidentiality.

## Results

### Participant flow and baseline characteristics

**Figure 1:**
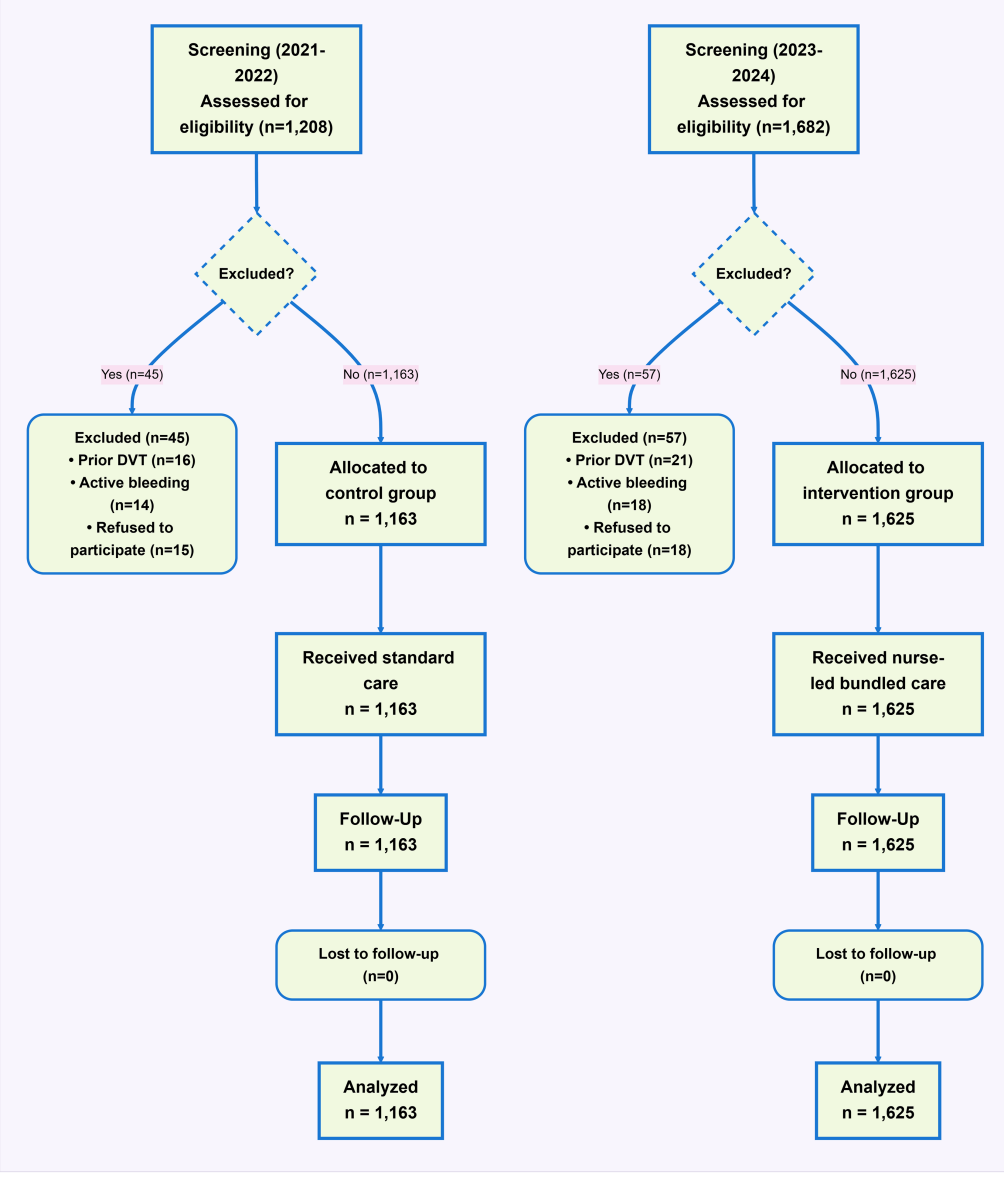
Flow diagram of participant selection and follow-up.

A total of 1,208 patients in the control group and 1,682 patients in the intervention group met the eligibility criteria. After exclusions (pre-existing DVT, active bleeding, refusal to participate) and loss to follow-up (transfer, early discharge), the final sample included 1,163 patients in the control group and 1,625 patients in the intervention group (Figure 1). There were no significant differences between the two groups in terms of age, sex, diagnosis, type of surgery, Caprini score, or reasons for immobilization (*p* > 0.05), indicating good baseline comparability (Table 1). In addition, the rates of pharmacological thromboprophylaxis, as well as the prescription of GCS and IPC, were comparable between groups.

**Table 1:** Baseline demographic and clinical characteristics of participants.

Characteristic	Control group (*n* = 1,163)	Intervention group (*n* = 1,625)	*p*-value
Age (years), mean ± SD	52.5 ± 14.7	53.2 ± 15.1	0.214
Sex, male, *n* (%)	676 (58.1)	951 (58.5)	0.827
Diagnosis, *n* (%)			0.418
Traumatic Brain Injury	349 (30.0)	504 (31.0)	
Brain Tumor	302 (26.0)	410 (25.2)	
Cerebrovascular Disease	256 (22.0)	374 (23.0)	
Spinal Cord Injury	186 (16.0)	244 (15.0)	
Other	70 (6.0)	93 (5.8)	
Type of surgery, *n* (%)			0.638
Cranial Surgery	791 (68.0)	1,105 (68.0)	
Spinal Surgery	267 (23.0)	382 (23.5)	
Non-surgical	105 (9.0)	138 (8.5)	
Caprini Score, mean ± SD	7.8 ± 2.3	7.9 ± 2.4	0.256
Caprini Risk Level, *n* (%)			0.352
Moderate (3–4)	140 (12.0)	195 (12.0)	
High (5–8)	640 (55.0)	878 (54.0)	
Highest (≥9)	383 (33.0)	552 (34.0)	
Immobilization Duration (days), mean ± SD	8.4 ± 3.6	8.2 ± 3.4	0.128
Pharmacological prophylaxis, n (%)	764 (65.7)	1,092 (67.2)	0.399
Type of pharmacological prophylaxis			0.682
LMWH (enoxaparin)	712 (61.2)	1,028 (63.3)	
UFH	52 (4.5)	64 (3.9)	
GCS prescribed, *n* (%)	791 (68.0)	1,365 (84.0)	<0.001
IPC prescribed, *n* (%)	442 (38.0)	1,284 (79.0)	<0.001

CS, graduated compression stockings; IPC, intermittent pneumatic compression; LMWH, low molecular weight heparin; UFH, unfractionated heparin.

In the control group, GCS and IPC were prescribed but without standardized application protocols, whereas in the intervention group they were applied as part of the bundled protocol.

### Primary outcome

Unadjusted comparisons showed that the incidence of symptomatic DVT was significantly lower in the intervention group compared to the control group. As shown in Table 2, DVT occurred in 27 patients (1.66%) in the intervention group versus 32 patients (2.75%) in the control group (*p* = 0.041). This represents a 39.6% relative risk reduction in DVT incidence following implementation of the nurse-led bundled care protocol.

**Table 2:** Comparison of primary and secondary outcomes between groups (unadjusted).

Outcome	Control group (*n* = 1,163)	Intervention group (*n* = 1,625)	Test statistic	*P*-value
Primary outcome				
Symptomatic DVT Incidence, *n* (%)	32 (2.75)	27 (1.66)	*χ*^2^ = 4.18	0.041
Secondary outcomes				
Compliance with GCS/IPC, *n* (%)	676 (58.1)	1,243 (76.5)	*χ*^2^ = 108.3	<0.001
Compliance with Mobilization, *n* (%)	599 (51.5)	1,172 (72.1)	*χ*^2^ = 125.7	<0.001
Post-Intervention Knowledge Score (Mean ± SD)	62.1 ± 10.2	85.2 ± 8.5	*t* = 66.4	<0.001
Limb Circumference Difference at Discharge (cm, Mean ± SD)	2.1 ± 1.1	1.2 ± 0.8	*t* = 25.3	<0.001
Post-Intervention VAS Pain Score (Mean ± SD)	4.1 ± 1.6	2.3 ± 1.2	*U* = 485,000	<0.001
Patient Satisfaction Score (Mean ± SD)	85.6 ± 4.8	94.1 ± 3.3	*t* = 56.1	<0.001
Pulmonary Embolism, *n* (%)	2 (0.17)	2 (0.12)	Fisher’s exact	0.715

### Secondary outcomes

Compliance and Knowledge: Patients in the intervention group demonstrated significantly higher compliance with both GCS/IPC use (76.5% vs. 58.1%, *p* < 0.001) and the early mobilization protocol (72.1% vs. 51.5%, *p* < 0.001). Their DVT-related knowledge scores at discharge were also significantly higher than those in the control group (85.2 ± 8.5 vs. 62.1 ± 10.2, *p* < 0.001).

Clinical Symptom Improvement: At discharge, patients in the intervention group had a significantly smaller between-leg circumference difference (1.2 ± 0.8 cm vs. 2.1 ± 1.1 cm, *p* < 0.001) and significantly lower VAS pain scores (2.3 ± 1.2 vs. 4.1 ± 1.6, *p* < 0.001) compared to the control group.

Patient Satisfaction and Safety Outcomes: The intervention group reported significantly higher overall satisfaction with nursing care (94.1 ± 3.3 vs. 85.6 ± 4.8, *p* < 0.001). The incidence of PE was very low in both groups (2 patients [0.12%] vs. 2 patients [0.17%], *p* = 0.715). No significant difference was found in the rates of bleeding (1.2% vs. 1.1%, *p* = 0.856) or skin complications (2.1% vs. 1.9%, *p* = 0.632).

After multivariable adjustment (Table 3), the bundled intervention remained independently associated with a 46% lower odds of DVT (adjusted OR = 0.54, 95% CI 0.32–0.92, *p* = 0.023). Sensitivity analysis excluding patients who received pharmacological prophylaxis (*n* = 752 in control, *n* = 533 in intervention) showed a consistent, although borderline, protective association (adjusted OR = 0.59, 95% CI 0.33–1.02, *p* = 0.059).

**Table 3:** Multivariable logistic regression analysis for symptomatic deep vein thrombosis.

Variable	Adjusted OR	95% CI	*P*-value
Group (Intervention vs. Control)	0.54	0.32–0.92	0.023
Age (per year)	1.01	0.99–1.03	0.289
Sex (Female vs. Male)	0.92	0.55–1.54	0.752
Diagnosis (reference: TBI)			
Brain tumor	1.15	0.58–2.28	0.687
Cerebrovascular disease	1.22	0.62–2.41	0.564
Spinal cord injury	1.48	0.71–3.09	0.294
Surgery type (ref: Cranial)			
Spinal surgery	1.35	0.72–2.53	0.349
Non-surgical	0.89	0.38–2.08	0.789
Caprini score (per point)	1.12	0.98–1.28	0.098
Pharmacological prophylaxis (Yes vs. No)	0.72	0.42–1.23	0.230
Calendar year (per year)	0.95	0.72–1.25	0.713

TBI, traumatic brain injury; OR, odds ratio; CI, confidence interval.

### Daily changes in lower limb circumference and pain scores

**Figure 2:**
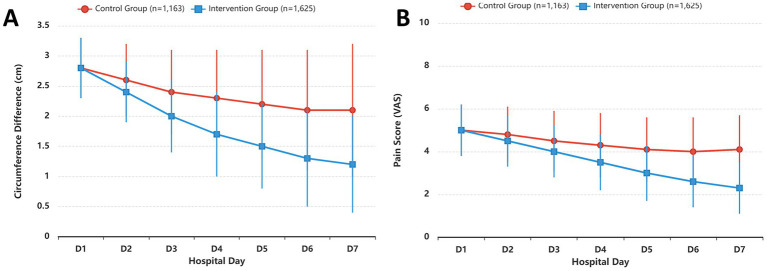
Daily changes during hospitalization **(A,B)**. Lower limb circumference **(A)** and pain scores **(B)**.

Figure 2A presents line chart showing the mean between-leg circumference difference (measured 10 cm above the patella) from day 1 to day 7 post-admission in the control group (*n* = 1,163, blue line with circles) and intervention group (*n* = 1,625, purple line with circles). The intervention group demonstrated a consistently greater and more rapid reduction in lower limb swelling throughout the hospitalization period. By day 3, a statistically significant separation between groups was observed (*p* < 0.05), which persisted through day 7. Error bars represent standard deviations. At discharge (day 7), the mean circumference difference was 1.2 ± 0.8 cm in the intervention group versus 2.1 ± 1.1 cm in the control group (*p* < 0.001). Figure 2B shows the daily changes in Visual Analogue Scale (VAS) pain scores over the same period. Baseline pain scores were comparable between groups (5.0 ± 1.2 in both). From day 2 onward, the intervention group exhibited consistently lower pain scores than the control group, with a significant separation emerging from day 3 (*p* < 0.05). By day 7, the mean VAS score in the intervention group was 2.3 ± 1.2, compared to 4.1 ± 1.6 in the control group (*p* < 0.001), reflecting a greater and sustained analgesic effect of the nurse-led bundled care protocol. Error bars represent standard deviations.

### Correlation between compliance with mechanical prophylaxis and DVT incidence

**Figure 3:**
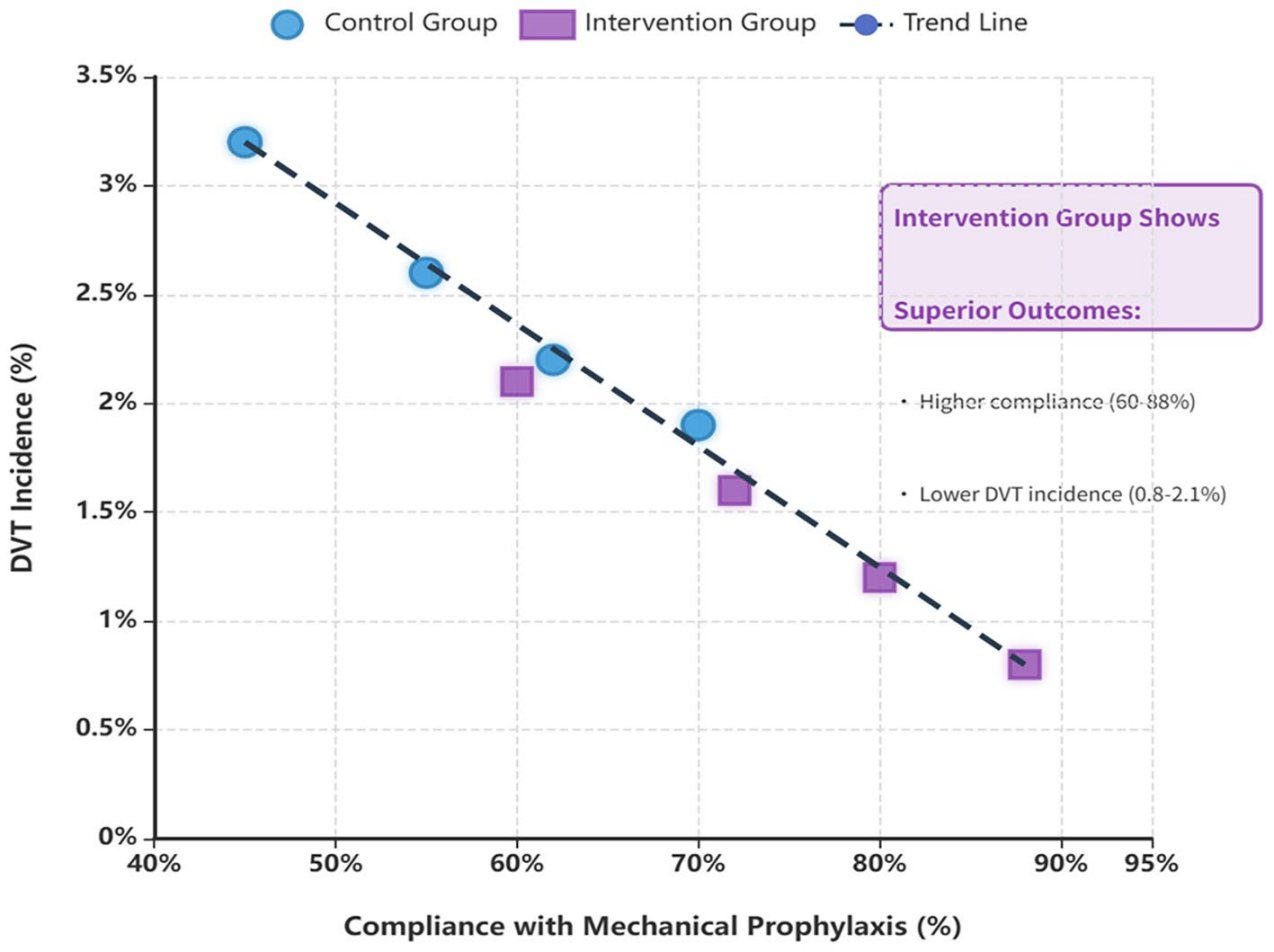
Correlation between compliance with mechanical prophylaxis and DVT incidence.

Figure 3 illustrates Correlation between compliance with mechanical prophylaxis and DVT incidence. In both the control (blue dots) and intervention (purple dots) groups, higher compliance with graduated compression stockings (GCS) and intermittent pneumatic compression (IPC) was associated with lower DVT incidence. The intervention group achieved both higher compliance rates and lower DVT incidence across all compliance levels, suggesting that the nurse-led bundle enhanced the effectiveness of mechanical prophylaxis through improved adherence. The dashed line represents the overall inverse correlation (*R*^2^ = 0.82, *p* < 0.001).

### Subgroup analysis of DVT risk reduction with nurse-led bundled care

**Figure 4:**
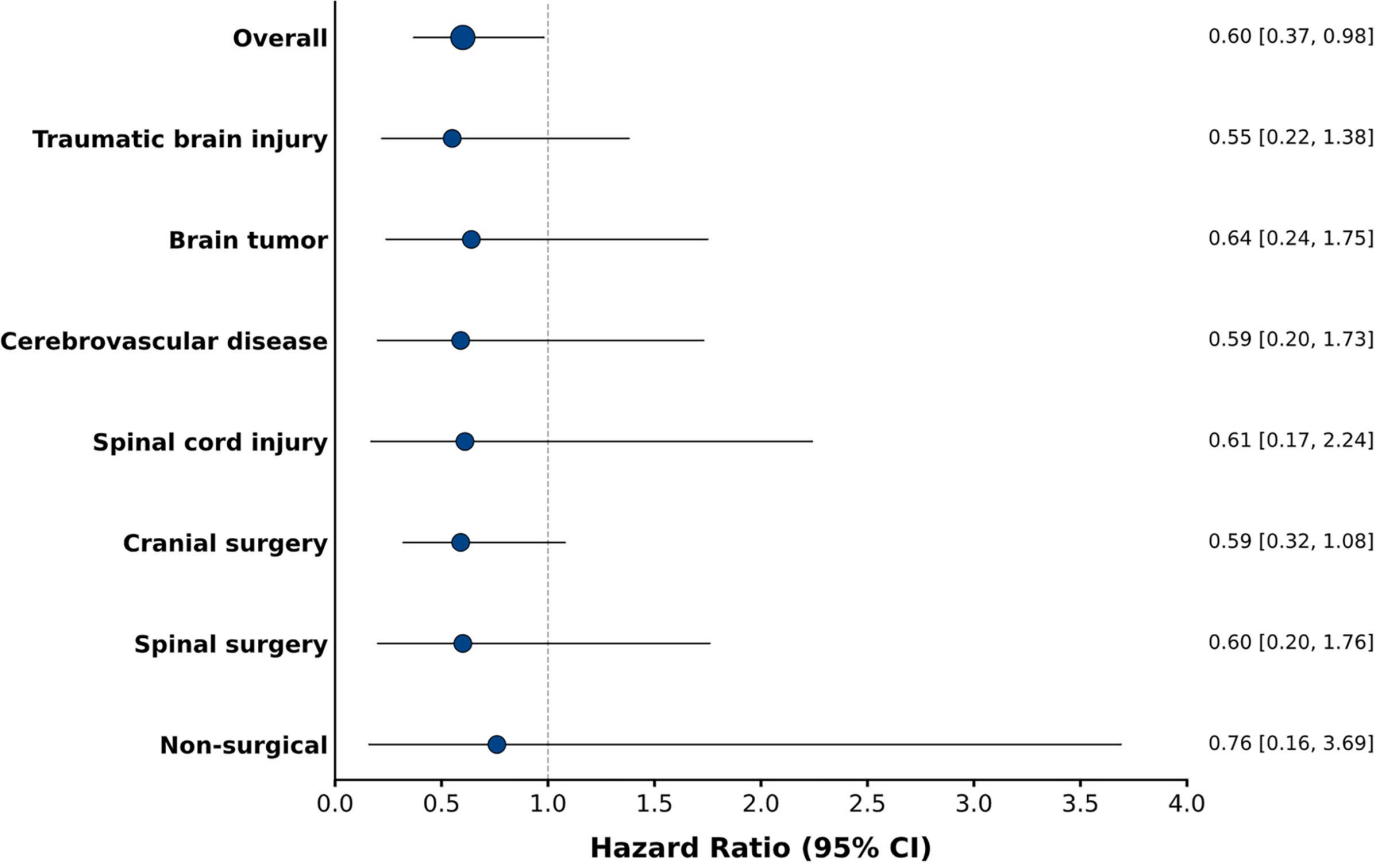
Subgroup analysis of DVT risk reduction with nurse-led bundled care. Forest plot displaying Mantel–Haenszel risk ratios (RR) and 95% confidence intervals (CI) for symptomatic DVT in the intervention group compared to the control group across predefined clinical subgroups. The overall RR was 0.60 (95% CI 0.37–0.98, *p* = 0.041), indicating a 40% relative risk reduction with the nurse-led bundle. No time-to-event analysis was performed. The vertical dashed line indicates the null value (RR = 1.0).

As shown in Figure 4, a forest plot displays the Mantel–Haenszel risk ratios (RRs) and 95% confidence intervals (CIs) for symptomatic DVT in the intervention group compared with the control group across predefined clinical subgroups. The overall RR was 0.60 (95% CI 0.37–0.98, *p* = 0.041), indicating a 40% relative risk reduction associated with the nurse-led bundle. Consistent protective effects were observed across all diagnostic and surgical subgroups, although confidence intervals widened in smaller subgroups. No significant heterogeneity was detected (*I*^2^ = 0%, P for interaction = 0.89). The vertical dashed line represents no effect (RR = 1.0).

## Discussion

This quasi-experimental study provides robust evidence that a nurse-led, multifaceted bundled care intervention is independently associated with a lower incidence of DVT in high-risk, immobilized neurosurgical patients. After adjusting for baseline characteristics and secular trends, patients who received the bundle had 46% lower odds of developing symptomatic DVT. The findings demonstrate that moving beyond single, fragmented preventive measures to a coordinated, evidence-based bundle yields substantial clinical benefits, with a 39.6% relative reduction in DVT incidence (from 2.75 to 1.66%, *p* = 0.041). Our observed DVT rate in the intervention group (1.66%) aligns favorably with contemporary international benchmarks; for instance, Chavarro et al. (12) recently reported that perioperative VTE incidence in neurosurgical patients receiving chemoprophylaxis at Ochsner Health System consistently ranges between 1.7 and 3.5%. This congruence suggests that our nurse-led bundle achieves outcomes comparable to those reported in well-resourced Western healthcare settings, despite potential differences in patient populations and healthcare delivery systems.

The success of this intervention likely lies in its multi-targeted approach, which addresses the complex pathophysiology of thrombosis in neurosurgical patients (14). Banerjee et al. (5), in their comprehensive review of VTE prophylaxis in the neurocritical intensive unit, emphasized that effective prevention requires simultaneous consideration of pharmacological and mechanical strategies, with careful attention to timing and patient-specific risk factors. The Caprini RAM ensured that preventive efforts were appropriately stratified, directing the most intensive resources to the highest-risk patients—88% of our patients were at high or highest risk (Caprini score ≥5), consistent with the inherent thrombotic risk in neurosurgical populations reported in previous study of bundled nursing strategies for cerebral hemorrhage patients (15). The Caprini score’s utility in this context is well-established. Researchers previously demonstrated its effectiveness when combined with nursing interventions in Chinese neurosurgical populations, showing significant reductions in DVT events through targeted risk stratification (13).

The combination of GCS and IPC addressed venous stasis through both passive (elastic compression) and active (simulated muscle pump) mechanisms, likely offering a synergistic effect greater than either alone (16). However, the optimal role of mechanical prophylaxis in the era of widespread pharmacological prevention warrants careful consideration. The ongoing GRACE trial (11), a large UK multicenter randomized controlled trial involving 8,608 surgical patients, is actively investigating whether GCS provide additional benefit when added to extended pharmacological thromboprophylaxis. While our study demonstrated significant benefits with combined mechanical prophylaxis, the GRACE trial’s results—expected to report in the coming years—may provide more definitive guidance on whether GCS remain necessary in patients already receiving optimal anticoagulation. Notably, the GRACE protocol highlights that two recent large trials in stroke and orthopedic patients failed to support GCS use, raising important questions about the incremental value of compression stockings. Our findings, which show substantially improved compliance (76.5% vs. 58.1%) and clinical outcomes with nurse-led mechanical prophylaxis implementation, suggest that even if the biological effect of GCS is modest, the structured nursing oversight may enhance overall prevention through improved adherence and monitoring. This interpretation aligns with the broader literature on implementation science, which emphasizes that intervention fidelity and patient adherence are critical determinants of real-world effectiveness.

Structured education empowered patients, transforming them from passive recipients into active partners in their own care, a factor increasingly recognized as critical for prevention success. Hongfang et al. (15) demonstrated that bundled nursing strategies incorporating comprehensive education significantly improved disease cognition, coagulation parameters, and quality of life in nonsurgical cerebral hemorrhage patients. Their finding that patient satisfaction scores improved from 0.865 (control) to 0.942 (intervention) parallels our observation of enhanced satisfaction (94.1 vs. 85.6), suggesting that the educational component may drive not only clinical improvements but also enhanced patient experience. The dramatic improvement in knowledge scores (from 62.1 to 85.2) in our study likely contributed to the observed compliance gains, as patients who understand the rationale for prophylaxis are more likely to adhere to sometimes uncomfortable interventions like GCS and IPC. This educational effect may be particularly important in Chinese healthcare settings, where traditional patient-provider relationships have often been paternalistic; our nurse-led model represents a shift toward patient-centered care that empowers patients through knowledge.

This study underscores the unique and powerful position of nurses in driving quality improvement in VTE prevention. As the consistent presence at the bedside, nurses were able to ensure the fidelity of the bundle’s implementation from initial risk assessment and daily application of mechanical devices to ongoing education and encouragement for early activity. Chavarro et al. (12) similarly emphasized that successful VTE prevention in neurosurgery requires systematic, protocol-driven approaches, noting that inconsistent recommendations from various societies have led to varied practices based on individual surgeon preference. Their quality improvement initiative at Ochsner Health System, implementing a department-wide chemical prophylaxis protocol, aims to reduce VTE incidence by 50%, *n* ambitious goal that our 39.6% relative risk reduction approaches. The nurse-led model fosters continuity of care and allows for real-time adjustments based on patient feedback and clinical status—advantages difficult to achieve with physician-ordered, siloed interventions. Researchers previously demonstrated the effectiveness of nurse-led VTE prevention bundles in UK hospital settings, reporting improved compliance and reduced VTE events through dedicated nursing leadership (17). Our findings extend this evidence to the Chinese context, demonstrating that nurse-led models are similarly effective across different healthcare systems. Notably, our intervention period (2023–2024) coincided with the hospital-wide increase in VTE prevention rates from 60.5 to 75.7%, suggesting that the neurosurgical nurse-led model may have contributed to and benefited from broader institutional quality improvement efforts, a synergistic effect that enhances the sustainability of such interventions.

For clinical practice, this study offers a clear, actionable, and standardized protocol readily adoptable by neurosurgical units. The bundle comprises inexpensive, widely available interventions (GCS, IPC, educational materials), making it highly feasible even in resource-constrained settings. Based on our hospital’s average cost for DVT management (approximately ¥15,000 per case), the prevention of an estimated 18 DVT cases in the intervention group (calculated as [2.75–1.66%] × 1,625) represents approximately ¥270,000 in direct cost savings, excluding additional savings from reduced length of stay and long-term complications such as post-thrombotic syndrome. Banerjee et al. (5) highlighted the substantial healthcare burden of VTE in neurocritical care unit, emphasizing that effective prevention strategies must balance efficacy, safety, and cost-effectiveness. The success in this high-risk neurosurgical population suggests that this nurse-led bundle model could be adapted for other immobilized patient populations (e.g., post-major orthopedic surgery, critical care, stroke units), offering a blueprint for hospital-wide VTE prevention programs.

This study has several limitations. First, the quasi-experimental design with a historical control group is susceptible to temporal bias, as changes in other aspects of care over time (e.g., increased use of anticoagulants, improved diagnostic capabilities) could have influenced results. Second, the single-center design may limit generalizability to settings with different patient populations or healthcare systems. Additionally, DVT was diagnosed only upon clinical suspicion, which likely underestimated the true incidence; the heightened awareness in the intervention group could theoretically have increased detection, meaning the observed reduction may be conservative. Future studies should incorporate protocolized screening ultrasound.

Although the quasi-experimental design limits causal inference, the consistent findings after multivariable adjustment and sensitivity analyses support an independent contribution of the nurse-led bundle. These results warrant confirmation in a multicenter randomized trial.

## Data Availability

The raw data supporting the conclusions of this article will be made available by the authors, without undue reservation.
